# The relationship between the ethical attitudes and holistic competence levels of intensive care nurses: A cross-sectional study

**DOI:** 10.1371/journal.pone.0287648

**Published:** 2023-07-13

**Authors:** Nihal Taşkıran, Gulengun Turk

**Affiliations:** Department of Fundamentals of Nursing, Aydın Adnan Menderes University College of Nursing, Aydın, Turkey; University Medical Centre Ljubljana (UMCL) / Faculty of Medicine, University Ljubljana (FM,UL), SLOVENIA

## Abstract

**Background:**

Intensive care units are directly associated with the competency level of nurses and are units where ethical problems are frequently experienced. This research aims to determine the relationship between intensive care nurses’ ethical attitudes and holistic competence levels.

**Methods:**

This study was conducted as a cross-sectional design using self-report questionnaires distributed to 131 intensive care nurses in Turkey. The data of the study were collected with the “Nurses Information Form,” “Holistic Nursing Competence Scale” and “Ethical Attitude Scale for Nursing Care”.

**Results:**

The total mean score of the Holistic Nursing Competence of the nurses was 6.89±0.95. Holistic Nursing Competence level was significantly lower for those who had experienced less than one year in the profession, and it was higher for those who worked in the emergency intensive care unit and the nurses whose clinics had 21 and above nurses. The total mean score of the nurses’ ethics attitude toward nursing care was 59.36±29.09. Ethical Attitude for Nursing Care was significantly lower for those who had a master’s degree, and the nurses whose clinics had 21 and above nurses scored higher. There was a weak and negative correlation between the nurses’ Holistic Nursing Competence Scale and the total mean score of the Ethical Attitude Scale for Nursing Care. The ethical attitude was predicted in 13.2% of the Holistic Nursing Competence of nurses.

**Conclusions:**

It was concluded that nurses’ holistic competence levels were high, their ethical attitudes were negative, and there was a weak negative correlation between their holistic competence levels and their ethical attitudes toward care.

## Introduction

Ethical attitude in nursing care is the internalization of professional, ethical principles and values and reflecting them in the behavior and care delivered to the patient [1, 2]. A moral process occurs between the nurse and the patient during caregiving [3]. The ethical attitude of the nurse in care practices is essential for the quality of care [4]. Nursing competence is the ability to effectively reflect a range of characteristics, such as the attitude, knowledge, and skills of a nurse, into care to fulfill their professional responsibilities, and it is a crucial attribute for assuring high-quality, ethical, and safe nursing care [5]. Nurses require to enhance their competencies to deliver the patient care expected from them in the most accurately, safely and ethicaly [6–8].

Nurses’ awareness of their ethical values and the effect of these values on their behavior is a core part of holistic and humanistic nursing care [9]. Moreover, one of the criteria for being a professional is to adopt professional, ethical principles in patient care. Nursing competency requires providing safe and high-quality nursing care by ethical principles. These ethical principles in nursing can be ranked as autonomy, beneficence, nonmaleficence, veracity, confidentiality, justice, and fidelity [10, 11].

Competency is a dynamic process that begins with vocational training and continues throughout working life. Benner defined competence as the ability to perform nursing tasks with the integration of knowledge to achieve desirable outcomes [12]. Meanwhile, the concept of "holistic competence" in nursing includes all nursing skills, as well as the nursing process, ethically oriented care, cooperation with team members, leadership, experience, intuition, critical thinking, knowledge level, education, and their professional roles and responsibilities [13–15]. In literature stated that nurses’ patient-centered care competence and holistic nursing competence levels were high [16–18]. Lu (2018) reported that holistic nursing practices improve the quality of care and satisfaction in patients [19]. Additionally, Aydın and Hicdurmaz (2019) stated that holistic nursing is an essential element of nursing care, as it enhances the quality of nursing care [20].

Intensive care units (ICUs) are clinics where ethical problems are common, such as using autonomy, inexperience about ethical problems, fairness in resource use, ethical decision-making, and giving information to the patient [3, 4, 21, 22]. Critical care nurses are at high risk for moral distress due to ethical conflicts created by technological advancement, high-intensity work environments, and frequent exposure to life and death decisions in the intensive care unit. Implicit rationing by nurses may reduce the standards of care offered, increasing the risk of adverse patient outcomes, threatening patient safety, reducing care quality, and directly contradicting ethical codes [23, 24]. Suhonen et al. (2018) stated that nurses attempt to prioritize nursing care based on a desire to satisfy all the needs of their patients holistically and comprehensively. However, when resources are low and with environmental constraints, nurses have difficulties fulfilling their professional, ethical roles [23]. A study conducted with intensive care nurses in Turkey concluded that 60.8% of the nurses experienced ethical dilemmas [25]. In a study, nurses considered leaving critical care because of moral distress [3]. The problems experienced may have a negative impact on the holistic competence levels of nurses by affecting the patient care behaviors of nurses in the ICU [26]. Nurses are responsible for making the best decisions for patients, and being a nurse requires assuming ethical responsibility in the ICU [27]. Holistic nurses should be experts in embodying nursing’s ethical tenets [28].

When the literature on the subject is reviewed, it is noticed that there are limited studies investigating the spiritual care competencies, essential competencies, holistic care perceptions, and ethical attitudes of intensive care nurses [16, 17, 29–31]. However, no study was found in which the ethical attitudes and holistic competencies of nurses were analyzed together, and the relationship between them was investigated. Holistic competence in care has a significant role in accelerating the recovery process in the ICU [31]. Since intensive care nurses face more ethical problems than in other nursing practice areas, the competence and ethical attitudes of nurses gain more significance in the ICU [3, 32].

Based on the STROBE checklist [S1 File], this research aims to determine the relationship between the ethical attitudes and holistic competence levels of intensive care nurses in a hospital in Turkey.

Research questions:

1. What are the holistic competency levels of intensive care nurses in Turkey?
2. What are the ethical attitudes levels of intensive care nurses in Turkey?
3. What is the relationship between the holistic competence levels and ethical attitudes of intensive care nurses?

## Methodology

### Design

This was a cross-sectional study employing self-report questionnaires.

### Setting and sample

The universe of the study consisted of 220 nurses who worked in intensive care units at a university hospital in Aydın, Turkey. These intensive care units were general, internal, general surgery, neurosurgery, cardiovascular surgery, thoracic, anesthesia and reanimation, emergency, coronary, and neurology intensive care. The “G. The Power-3.1.9.2” program was used to estimate the appropriate number of nurses needed for a study sample with reference to Pai (2015) [33]. It was calculated through power analysis that a sample size must include at least 121 nurses (p<0.05 significance) to provide an 80% theoretical power with 95% confidence and 0.33 effect size.

131 nurses above 18 years old who worked in intensive care for at least six months, offered adult patient care, and consented to participate were included in the present study.

This study conducted self-administered questionnaires between June and December 2021 at a university hospital in Aydın, Turkey. The questionnaire forms included no information on the identification of the participants.

### Data collection tools

Data for the current study were collected utilizing the “Nurses Information Form,” “Holistic Nursing Competence Scale” and “Ethical Attitude Scale for Nursing Care” [S2 File]. It took approximately 25 minutes for nurses to fill out the questionnaires.

### Nurses Information Form

This form was developed by the researchers in line with the literature to determine their demographic characteristics [4, 34, 35]. The form contained 13 questions that investigated the sociodemographic characteristics of the nurses (e.g., age, gender, marital status, educational level, professional status, and working time).

### Holistic Nursing Competence Scale (HNCS)

The HNCS is a 7-point Likert-type scale that was developed by Takase & Teraoka [36]. It comprises two parts, five subscales, and 36 items in total. The first part (A) involves only the ’General Aptitude’ subscale of the HNCS, which consists of questions related to general behaviors as a person rather than as a nurse. This part comprises seven questions in total, and the items are scored from 1 (never) to 7 (always). The second part (B) measures the competence of a nurse and consists of the remaining four subscales of the HNCS, which are as follows: ’Staff Education and Management’, ‘Ethically Oriented Practice’, ‘Nursing Care in Team’ and ‘Professional Development’. This part (B) consists of 29 items that are scored from 1 (not competent at all) to 7 (very competent). The scale contains no inversely scored item or cutoff point. The final HNCS score is ascertained from the simple addition of the subscale scores [36]. There is no grading in the evaluation of the scale. A high total mean score on the scale indicates high nursing competency. The Turkish adaptation of the scale was performed by Saldiroglu and Turk [35]. The Cronbach’s alpha internal consistency coefficient of the scale was 0.98, and in the present study, the total scale item internal consistency coefficient was calculated as 0.98.

### Ethical Attitude Scale for Nursing Care (EASNC)

This scale was developed by Ozciftci and Akın (2020) to determine the ethical attitudes of nurses during care [34]. The scale has 34 items and uses a five-point Likert scale, where participants scored from 1 “strongly disagree” to 5 “strongly agree.” Possible scores range from 34 to 170. A high score on the scale indicates a positive ethical attitude. The Cronbach’s alpha internal consistency coefficient of the scale was 0.96, and in the present study, the total scale item internal consistency coefficient was calculated as 0.99.

### Data collection

The nurses who agreed to participate in the study completed the self-administered questionnaires between June and December 2021. Those working in a hospital were invited to take part in the survey on a voluntary basis upon permission from the head of the hospital and the nurse managers. All nurses working in the ICUs were given the questionnaires by the researchers. Each of the participants was given a questionnaire form along with an envelope and a letter explaining the complete research study, the instructions for filling out the questionnaire, and written assurance stating that participation was voluntary. After filling out the questionnaires, the participants put them in envelopes and gave them to the nurse manager. Following this, the researchers obtained the questionnaires from the nurse manager.

### Data analysis

To perform the statistical analysis for the present study, the authors utilized the Statistical Package for Social Science (SPSS 22.0) software program. The descriptive statistics were provided in a number and percentage format, and the skewness and kurtosis values were used to examine the normal distribution of variables. Between -2 and +2 values were accepted as a normal distribution [37]. The comparison between the nurses’ profile and key variables of the present study was analyzed using the Mann‒Whitney U or independent-samples t test for binary groups and the Kruskal‒Wallis or one-way ANOVA test for triple groups. The correlation between holistic nursing competence and ethical attitudes toward nursing care was analyzed by Pearson’s rho coefficients. Linear regression was used to assess the predictive power of the variables of the study. Statistical significance was accepted at p<0.05.

### Ethics approval and consent to participate

We confirm that all procedures utilized in this study were conducted following the principles of the Helsinki Declaration. Ethical clearance and approval letters were secured from the Institutional Review Board (IRB) of the Aydın Adnan Menderes University of Nursing Ethics Committee (Approval Number: 2021–246). All nurses agreed to voluntarily participate in the present study, verbal consent was obtained from all of them, and they were informed about the purpose of this study. During the data collection, the issue of confidentiality and privacy was assured by maintaining the anonymity of participants. Participants could withdraw from the study at any time if they were not happy with the survey. Written approval was obtained from hospital administrators to carry out the study. The necessary permission to utilize the scales discussed previously in this study was received from the authors.

## Results

The average age of the nurse participants was 28.22±4.44, most of them (81.7%) were female, and more than half of the nurses were single (51.9%) and had a bachelor’s degree (67.9%). The mean years of working experience of the nurses was 2.67±0.82 (Median = 3.00) years, and the mean working intensive care experience was 2.29±0.75 (Median = 2.00) years. Approximately a quarter of the nurses (19.8%) were working in the internal intensive care unit. A majority of the nurses (69.5%) reported that they worked with a total of 16–20 nurses in their clinics. Additionally, almost all of the nurses (90.1%) gave care to 1–3 patients for each shift. Most of the nurses (87%) reported that they had received training on ethics (Table 1).

**Table 1:** Descriptive features of nurses.

Descriptive Features (n = 131)	n	%
**Gender**		
Female	107	81.7
Male	24	18.3
**Marial status**		
Single	68	51.9
Married	63	48.1
**Education**		
Master degree	9	6.9
Bachelor degree	89	67.9
Associate degree	18	13.7
Medical vocational high school*	15	11.5
**Professional experience**		
<1 year	7	5.3
1–5 years	52	39.7
6–10 years	49	37.4
≥11 years	23	17.6
**Experience of intensive care unit**		
6 month-1 year	18	13.7
2–5 years	61	46.6
6–10 years	47	35.9
>11 years	5	3.8
**Intensive care unit**		
Emergency	5	3.8
Coronary	5	3.8
Anesthesia and reanimation	7	5.3
General	8	6.1
General surgery	9	6.9
Thoracic	12	9.2
Cardiovascular surgery	19	14.5
Neurology	19	14.5
Brain surgery	21	16.0
Internal	26	19.8
**Number of nurses working in the intensive care unit**		
5–10 nurses	5	3.8
11–15 nurses	23	17.6
16–20 nurses	91	69.5
≥21 nurses	12	9.2
**Number of patients caring in a shift**		
1–3 patients	118	90.1
4–6 patients	4	3.1
7–9 patients	5	3.8
≥10 patients	4	3.1
**Course on ethics**		
Yes	114	87.0
No	17	13.0
**Mean Age: 28.22±4.44 years**		

*Medical vocational high school: 4-year high school, after an 8-year primary school.

The total mean score of the HNCS of the nurses was 6.89±0.95, and the total mean scores of the subscale for "General Aptitude" was 5.53±0.90, for “Staff Education and Management” was 5.36±0.94 and for “Ethically oriented practice and professional development” was 5.64±0.80. The total mean score of the EASNC of the nurses was 59.36±29.09 (Table 2).

**Table 2:** Descriptive statistics and indicators related to scales.

	HNCS	HNCS Sub-scales			EASNC
	Total	General Aptitude	Staff Education and Management	Ethically oriented practice and professional development	Total
n	131	131	131	131	131
Number of scale items	36	7	9	20	34
Minimum-maximum	4.45–8.69	3–7	2.56–7	2.45–7	34–170
Mean±SD	6.89±0.95	5.53±0.90	5.36±0.94	5.64±0.80	59.36±29.09
Cronbach’s alpha coefficient	0.98	0.95	0.96	0.98	0.99
Skewness±SD	-0.203±0.21	-0.426±0.21	0.541±0.21	0.539±0.21	2.025±0.21
Kurtosis±SD	0.405±0.42	-0.148±0.42	0.397±0.42	0.915±0.42	4.791±0.42

There was a weak and negative correlation between the nurses’ HNCS and the total mean score of the EASNC (r = -0.363, p = 0.000). The results revealed that ethics attitude toward nursing care negatively affects nurses’ holistic nursing competence (β = -0.363, p = 0.000) (Table 3). The linear regression model revealed that ethical attitude for nursing care could explain 13.2% of the holistic competence of nurses (R^2^ = 0.132).

**Table 3:** Effect of the ethic attitude for nursing care on holistic nursing competence of nurses.

Unstandardized Coefficients			Standardized Coefficients	t	p	95.0% CI
	B	SE	β			
Constant	220.493	5.162		42.714	0.000	210.280 to 230.706
EASNC	-0.346	0.078	-0.363	-4.425	**0.000** ^*^	-0.500 to 0.191
Dependent Variable: HNCS						
Durbin-Watson = 1.919 F = 19.576, p = 0.000						
R = 0.363, R^2^ = 0.132, Adjusted R^2^ = 0.125, * = p<0.05						

β: Standardized regression coefficient, CI: Confidence interval

EASNC: Ethic Attitude for Nursing Care, HNCS: Holistic Nursing Competence

The total mean score of the HNCS was significantly lower for nurses who had less than one year of experience in the profession (p<0.05). The HNCS mean scores of nurses who worked in internal intensive care were significantly lower than those of nurses who worked in the emergency intensive care unit (p<0.05). On the other hand, the HNCS mean scores of the nurses, where their clinics had 21 and above nurses in total, were significantly higher (Table 4). Gender, marital status, education level, experience in the intensive care unit, professional status, and number of patients caring in a shift did not have a statistically significant effect on the level of holistic nursing competence (p>0.05). The subscale “General aptitude” mean score was significantly higher for those who had a master’s degree. The “Staff education and management” subscale mean score was significantly higher for those who had more than 11 years of experience in professional and intensive care.

**Table 4:** Comparison of nurses HNCS with EASNC scores according to descriptive features.

	HNCS	HNCS Dimensions			EASNC
Descriptive Features (n = 131)	Total Score Mean±SD	General Aptitude Mean±SD	Staff Education and Management Mean±SD	Ethically oriented practice and professional development Mean±SD	Total Score Mean±SD
**Gender**					
Female	6.91±095	5.58±0.87	5.36±0.95	5.65±0.79	57.71±26.63
Male	6.79±0.99	5.29±1.02	5.33±0.93	5.60±0.86	66.70±38.04
Test value	0.547^a^	-0.998^b^	-0.033^b^	0.310^a^	-0.319^b^
p	0.586	0.318	0.974	0.757	0.750
**Marial status**					
Single	6.82±1.03	5.49±0.91	5.29±1.04	5.58±089	62.30±32.87
Married	6.97±0.86	5.57±0.90	5.43±0.82	5.72±0.70	56.19±24.24
Test value	-0.939^b^	-0.584^b^	-0.835^b^	-6.685^b^	-1.051^b^
p	0.349	0.559	0.404	0.494	0.293
**Education**					
Master degree	7.08±0.65	6.01±0.44	5.25±1.10	5.79±0.45	43.88±13.52
Bachelor degree	6.94±1.02	5.57±0.89	5.43±1.01	5.66±0.86	59.39±29.90
Associate degree	6.97±0.76	5.56±0.94	5.32±0.80	5.77±0.59	58.83±30.96
Medical vocational high school*	6.40±0.74	4.95±0.96	5.01±0.52	5.29±0.75	69.13±26.78
Test value	6.647^c^	3.114^d^	0.909^d^	1.231^d^	8.970^c^
p	0.084	**0.029**	0.439	0.301	**0.030**
**Professional experience**					
<1 year	5.83±0.63	5.24±0.85	3.93±0.97	4.85±0.51	57.28±17.08
1–5 years	6.88±0.98	5.50±0.99	5.36±1.00	5.64±0.78	57.65±27.65
6–10 years	7.00±0.87	5.51±0.91	5.47±0.81	5.76±0.72	59.83±32.01
≥11 years	6.99±0.98	5.73±0.70	5.54±0.74	5.64±0.99	62.86±29.89
Test value	3.368^d^	0.628^d^	6.546^d^	2.728^d^	1.404^c^
p	**0.021**	0.598	**0.000**	**0.047**	0.705
**Experience of intensive care unit**					
6 months-1 year	6.40±1.04	5.46±0.94	4.56±1.25	5.31±0.85	60.11±31.89
2–5 years	6.92±0.88	5.48±0.98	5.46±0.86	5.66±0.69	55.27±22.48
6–10 years	7.07±0.92	5.60±0.83	5.52±0.82	5.80±0.78	63.12±33.25
>11 years	6.60±1.32	5.68±0.54	5.40±0.46	5.16±1.60	71.20±47.92
Test value	2.404^d^	0.216^d^	5.437^d^	2.359^d^	1.508^c^
p	0.071	0.885	**0.002**	0.075	0.680
**Intensive care unit**					
Emergency	8.01±0.35	6.28±0.44	6.62±0.47	6.44±0.42	38.80±8.22
Coronary	7.22±0.61	5.77±0.43	5.60±0.60	5.94±0.63	48.20±18.11
Anesthesia and reanimation	6.65±0.58	5.38±0.70	5.33±0.40	5.36±0.60	53.00±14.73
General	7.18±0.85	5.83±0.94	5.69±0.78	5.80±0.65	51.87±17.38
General surgery	7.38±0.74	5.93±0.89	5.76±0.69	6.03±0.55	61.44±42.75
Thoracic	6.88±1.04	5.52±1.22	5.57±0.94	5.54±0.79	56.25±21.93
Cardiovascular surgery	6.53±0.77	5.27±0.73	5.25±0.75	5.26±0.82	77.10±40.65
Neurology	7.00±1.09	5.62±0.99	5.53±0.87	5.69±0.94	56.21±31.76
Brain surgery	6.99±0.96	5.66±0.96	5.13±1.07	5.85±0.71	57.57±17.69
Internal	6.53±1.00	5.16±0.81	4.88±1.08	5.46±0.86	61.00±29.02
Test value	2.151^d^	1.504^d^	2.612^d^	1.875^d^	10.516^c^
p	**0.030**	0.154	**0.009**	0.062	0.310
**Number of nurses working in the intensive care unit**					
5–10 nurses	6.33±0.78	4.92±0.80	5.19±0.34	5.11±0.95	49.00±17.57
11–15 nurses	6.84±0.97	5.48±0.91	5.26±0.97	5.63±0.81	50.34±29.53
16–20 nurses	7.06±0.55	5.93±0.81	5.51±0.68	5.69±0.45	60.76±27.81
≥21 nurses	7.34±0.85	5.94±0.61	5.79±1.01	5.95±0.64	70.33±38.12
Test value	3.363^d^	3.967^d^	2.135^d^	2.990^d^	7.845^c^
p	**0.021**	**0.010**	0.099	**0.034**	**0.049**
**Number of patients caring in a shift**					
1–3 patients	7.18±0.96	5.64±1.07	5.37±0.97	6.10±0.66	60.18±30.17
4–6 patients	6.92±0.50	5.53±0.92	5.37±0.44	5.73±0.42	47.25±16.47
7–9 patients	6.89±0.98	5.42±0.88	5.30±0.35	5.64±0.82	54.40±16.08
≥10 patients	6.46±0.50	5.40±0.47	5.19±1.11	5.08±0.49	53.50±16.36
Test value	0.385^d^	0.073^d^	0.048^d^	1.079^d^	0.999^c^
p	0.764	0.974	0.986	0.360	0.801
**Course on ethics**					
Yes	6.93±0.98	5.58±0.91	5.38±0.98	5.67±0.82	59.83±30.48
No	6.66±0.73	5.19±0.76	5.23±0.67	5.48±0.65	55.73±14.67
Test value	-1.812^a^	1.667^a^	0.594^a^	0.876^a^	-1.288^b^
p	**0.022**	0.098	0.554	0.383	0.198

^a^Independent-samples t test, ^b^Mann-Whitney U test, ^c^Kruskal-Wallis test, ^d^One-way ANOVA test, #Tukey test

The total mean score of the nurses at EASNC was significantly lower who had a master’s degree, and the nurses whose clinics had 21 and above nurses scored higher (p<0.05) (Table 4). Gender, marital status, experience in the profession and intensive care unit, professional status, working intensive care unit and shift, working times within night shift, and taking a course on ethics did not have a statistically significant effect on the ethical attitude for nursing care (p>0.05).

## Discussion

This study investigated the relationship between the ethical attitudes and holistic competence levels of intensive care nurses at a university hospital in Turkey. Evaluating the competence of nurses is essential to determine professional development and training needs and provide the best nursing care. Our study showed that the holistic nursing competence levels of nursing were high. In literature stated that nurses holistic competence levels of nursing were high [16–18]. Our study findings are in parallel with the findings of a limited number of studies on the subject. Lu (2018) reported that holistic nursing practices improve the quality of care and satisfaction in patients [19]. Additionally, Aydın and Hicdurmaz (2019) stated that holistic nursing is an essential element of nursing care, as it enhances the quality of nursing care [20]. The high level of holistic competence of the nurses in our study suggests that they provide care with a holistic approach to intensive care patients.

It is of great importance to adopt ethical attitudes, professional, ethical principles, and values in nursing care in ICUs and reflect this on behavior and patient care [3]. Ethical tensions, particularly those related to death and dying and life-sustaining interventions, frequently occur in the intensive care unit. Patients in intensive care units require a high level of nursing care for reasons such as their clinical course, changes in consciousness level, and being bedridden. For this reason, intensive care nurses may encounter situations that require urgent and critical decisions more frequently. Unresolved ethical conflict can lead to nurse moral distress and burnout, which may subsequently influence the care of patients [38]. Our study showed that nurses’ ethical attitude levels were low. In a study, nurses stated that they were insufficient in acting in accordance with ethical principles in patient care [39]. In another study, the moral sensibility of intensive care nurses in the study was moderate [40]. In another study, nurses estimated their own ethical competence to be at an average level [41]. Our study findings are in line with the literature [39, 42, 43]. The negative (low levels ethical attitude) ethical attitudes of the nurses who participated in our research may have resulted from the complex ethical problems experienced due to the critical importance of the patient group provided care for and the high workload and stress of the nurses. It is thought that this situation may directly affect the quality of nursing care to be delivered to intensive care patients. As of 2020, 198,465 nurses are serving in Turkey. Accordingly, the number of nurses per 1000 people is approximately 2.4, which is 70% below the OECD countries [44]. The high number of patients per nurse may lead to burnout for nurses working with intensive care patients, whose care is challenging and stressful. It is also considered that the burnout experienced may adversely affect nurses’ ability to make ethical decisions in patient care.

The ICU is an ethically charged environment: life and death decisions are made daily, in acute, highly emotional situations that often involve legally incompetent patients and their family. This responsibility requires the nurse to have a high level of competence [21, 45, 46]. A competent nurse can develop a more effective solution skill at the stage of assessing the existing ethical problem [15]. It is important to note that in our study, a 13.2% correlation was found between ethical attitudes and holistic competencies of nurses, although it was negative. However, it is suggested that a high level of competence has a favorable impact on developing a positive ethical attitude in intensive care nurses [15, 47]. It is thought that the negative correlation between ethical attitude and holistic competence in our study, contrary to the existing data in the literature, may have resulted from the fact that most nurses have low levels of ethical attitudes.

In our study, although the number of nurses with less than one year of professional experience was considerably low, it was found that these nurses had low holistic competency levels. It is reported in the literature that there is a positive correlation between the duration of professional experience and the level of nursing competency [48, 49]. A requirement of critical care registered nurse credentialing calls for at least two years of experience practicing in adult intensive care unit areas [50]. Our study finding is similar to the findings in the literature and supports the positive correlation between the duration of professional experience and the level of holistic competency.

The skills that a nurse needs to be qualified may vary depending on the field in which the nurse works. ICUs are clinics where nurses can exercise their autonomy at the highest level and thus reflect their competencies in patient care [51]. In our study, it is noteworthy that emergency intensive care nurses have the highest competency level, whereas the mean score of ethical attitudes is the lowest, although it is not significant. Fırat et al. (2017) reported that in the emergency department, nurses’ ethical sensitivity was moderate [52]. Factors such as the urgent and critical conditions of patients in emergency care and the high number of complex cases allow intensive care nurses to gain experience and improve their competence levels, but they also cause various ethical problems [52]. The low ethical attitude levels of nurses can be associated with their lack of time to solve ethical problems and the need for quick decision-making in the emergency ICU.

Likewise, in our study, it was revealed that both the holistic competence levels and ethical attitudes of the nurses in the ICUs, where the number of patients per nurse is low, are significantly higher. This finding is expected. It is considered that the low number of patients per nurse positively affects nurses’ ability to provide care with holistic competence and to make ethical decisions.

The results of the studies on the impact of education on ethical sensitivity and ethical attitudes in nurses show variability. In the studies of Yilmaz et al. (2018) and Kırca and Özgönül (2020), no significant difference was found between the education levels of nurses and their ethical sensitivity levels [53, 54]. Meanwhile, in the study of Kahriman and Yesilcicek-Çalık (2017), it was suggested that as the education level of nurses increased, ethical sensitivity increased [55]. In the same study, it was also determined that the ethical sensitivities of nurses who received training on ethics were lower than those who did not receive training. On the other hand, in the study of Yılmaz et al. (2018), it was found that receiving a course on nursing ethics did not make a positive contribution to ethical sensitivity [53]. In our study, although the majority of nurses reported that they received training on ethics, no significant correlation was found between education and ethical attitude. However, in our study, the ethical attitudes of nurses with a master’s degree were significantly negative. Saberi et al. (2019) found that nurses with a master’s degree were exposed to more ethical conflicts than other nurses [56]. In our study this result may be because only 6.9% of the nurses in the sample had a master’s degree and because the majority of the nurses with a master’s degree had insufficient professional experience.

### Limitations

The limitations of the present study include the fact that the nurses’ holistic competence levels and ethics attitudes were questioned via the questionnaire given, that the results of the study were based on the nurses’ self-reports and that no observational assessment was carried out. Since the current study was conducted at only one hospital in Turkey, these findings cannot be generalized.

## Conclusion

Based on our research findings, it has been concluded that nurses have high holistic competence levels but negative ethical attitudes, and there is a weak negative correlation between nurses’ holistic competence levels and their ethical attitudes. Holistic competence and ethical attitudes increase as the number of patients per nurse decreases and as professional experience and education increase.

It is recommended to periodically organize practical training that will contribute to the positive development of the ethical attitudes of intensive care nurses, providing ethical consultation when the nurses’ needs and assessing the holistic competencies and ethical attitudes of nurses at regular intervals. The reasons for the low ethical attitudes of nurses should be examined in future studies.

## Supporting information

S1 File STROBE checklist. (DOCX)

S2 File Data set. (SAV)
